# Changes in Cognitive Function and Risk Factors for Cognitive Impairment of the Elderly in China: 2005–2014

**DOI:** 10.3390/ijerph16162847

**Published:** 2019-08-09

**Authors:** Qilin Zhang, Yanli Wu, Tiankuo Han, Erpeng Liu

**Affiliations:** Centre for Social Security Studies, Wuhan University, Wuhan 430072, China

**Keywords:** Chinese elderly, cognitive function, changes, cognitive impairment, risk factors

## Abstract

*Background:* The cognitive function of the elderly has become a focus of public health research. Little is known about the changes of cognitive function and the risk factors for cognitive impairment in the Chinese elderly; thus, the purposes of this study are as follows: (1) to describe changes in cognitive function in the Chinese elderly from 2005–2014 and (2) to explore risk factors for cognitive impairment of the Chinese elderly. *Design and setting:* A total of 2603 participants aged 64 years and above participated in the Chinese Longitudinal Healthy Longevity Survey (CLHLS) and were followed up from 2005 to 2014. Cognitive function and cognitive impairment were assessed using the Chinese version of the Mini-Mental State Examination (MMSE). Binary logistic regression analysis was used to estimate the odds ratio (OR) and 95% confidence intervals (CI) of cognitive impairment. *Results:* Results revealed that the cognitive function of the Chinese elderly shows diversified changes: deterioration (55.09%), unchanged (17.21%) and improvement (27.70%). In addition, there are significant demographic differences in gender, age, education, marriage and other aspects when it comes to the changes of cognitive function in Chinese elderly. In the binary logistic regression analysis, female, increased age, lower education level, no spouse, less income, worse PWB (psychological well-being), less fresh fruit and vegetable intake, more activities of daily living (ADL) limitations, lower social engagement were significantly associated with higher odds for cognitive impairment. *Conclusions:* Various interventions should be implemented to maintain cognitive function in Chinese elderly.

## 1. Introduction

Aging is an important public health issue throughout the world, especially in China. By 2018, the number of Chinese elderly people over the age of 60 had reached 249 million, accounting for 17.9% of the total population [1]. It is estimated that by 2050, the number of elderly people over the age of 60 will exceed 450 million in China, representing over 30% of the total population [2]. With increasing population aging due to improvements in life expectancy, there will be a large population of older adults with a high risk for cognitive impairment [3,4]. Elderly people with mild cognitive impairment may experience cognitive dysfunction, while those with severe cognitive impairment may progress to dementia or Alzheimer’s disease (AD), thus losing the capacity to accomplish their daily routines and resulting in the inability to live independently. Cognitive decline is considered one of the most frightening aspects of aging [5,6]. At the end of 2013, the total number of patients in China with cognitive impairment reached 8.18 million. With an annual increase of more than 0.36 million, the total number of patients with cognitive impairment is expected to reach 48.68 million by 2060. China has become the fastest growing country in the world for people with cognitive impairment [7,8]. Cognitive impairment is a crucial health problem due to its adverse effect on both elderly people’s independent living and the heavy economic burden to homes and social care [9,10]. However, in China current research on changes in cognitive function and the risk factors for cognitive impairment of the elderly is scarce, as are policies concerning interventions for the prevention, diagnosis, treatment and support of cognitive impairment, which is extremely incompatible with a large-scale population with cognitive impairment.

Previous studies concerning cognitive function of the elderly have predominantly focused on detecting cognitive impairment and assessing cognitive decline in those with dementia [11,12,13,14]. To provide basic and relevant information for policy, program, and service development to support the public in maintaining cognitive function, experts in the field have called for taking “a more complete view of the nature and extent of cognitive aging in older adults” [15]. Some studies have explored changes in cognitive function of the elderly [16,17,18,19,20], but they were all focused on older adults in developed countries. To our knowledge, only a few studies have explored cognitive changes of the elderly in China [21,22,23]. These studies have enriched the academic field on cognitive function of the elderly in China; however, to be fair, their observations are only based on cross-sectional data regarding cognitive function in the elderly with a relatively limited coverage of samples in only a few provinces. Furthermore, there are some gaps in the research that remain to be filled, including differences in cognitive function change by gender, age, marriage status, education and place of residence.

As China, the world’s largest developing country, quickly transitions into an aging society, the prevalence and disease burden of cognitive impairment will be on the rise [7,24]. In addition to exploring the entire picture of changes in cognitive function of the elderly in China, this study also explores risk factors for cognitive impairment in the Chinese elderly. Risk factors for cognitive impairment have been extensively documented throughout the world and include: (1) demographics, such as age, gender, education level, marital status, coresidence and income [25,26,27,28,29,30]; (2) psychological factors [31,32]; (3) lifestyle, including diet, smoking, drinking and exercising [33,34,35,36,37,38]; (4) ADL [39,40]; (5) social engagement [41,42]; and (6) cardiovascular risk factors, including hypertension, diabetes, heart disease, stroke or CVD and dyslipidemia [43,44,45]. A few studies have attempted to identify risk factors for cognitive impairment of the Chinese elderly; however, the data were either too outdated or cross-sectional to precisely grasp the core of the issue from a dynamic perspective [23,46,47]. In addition, a previous study of the risk factors for cognitive impairment of Chinese elderly was scattered, needing to be integrated into a framework to analyze from multiple aspects to improve efficiency of the corresponding intervention measures [21,22,23]. The objectives of this study were two-fold: (1) to explore the changes in cognitive function among Chinese elderly 64 years of age and above using 10 years data from the CLHLS; and (2) to analyze risk factors for cognitive impairment in a framework from multiple aspects.

## 2. Materials and Methods

### 2.1. Participants and Settings

The data used in this study were derived from the CLHLS, and the survey was jointly performed by the Center for Healthy Aging and Development Studies at Peking University and Duke University. The CLHLS aims to understand the health status and associated social, behavioral, and biological factors among Chinese elderly. As the currently largest survey in the world in terms of health and longevity, a baseline survey was conducted in 1998, followed by six waves of surveys in 2000, 2002, 2005, 2008, 2011 and 2014 from 22 sample areas in 31 provincial administrative units (Beijing, Tianjin, Hebei, Shanxi, Liaoning, Jilin, Heilongjiang, Shanghai, Jiangsu, Zhejiang, Anhui, Fujian, Jiangxi, Shandong, Henan, Hubei, Hunan, Guangdong, Guangxi, Chongqing, Sichuan, Shanxi, and Hainan), which constituted 85% of the total population and covered the eastern, middle and western regions, as well as northeast China. The survey randomly selected around one-half of the counties in the 22 provinces as primary survey units [48,49,50]. The initial survey, participants were who aged 80 years and above in 1998 were followed in subsequent waves 2 to 3 years apart. In the CLHLS 2002 wave, an additional sample of adults 65 to 79 years of age was added to the survey, and some adults aged 64 were also included in the 2005 wave. Additional details, such as the sampling design and assessment of data quality, can be found at http://www.icpsr.umich.edu/icpsrweb/ NACDA/studies/36179. We use CLHLS data from four waves 2005–2014. Due to the dropouts and deaths of the elderly in the four waves follow-up survey, a total of 15638 CLHLS participants 64 years of age above in 2005, 16540 participants in 2008, 9765 participants in 2011 and 7192 participants in 2014. For merging data longitudinally from four waves, ID (identity code) was the only matching condition, after filtering for missing values, outliers, etc., data from 2603 elderly people aged 64 and above who participated in the survey in 2005 to 2014 were retained. Balanced panel data was subsequently constructed accordingly.

### 2.2. Measurement

#### 2.2.1. Cognitive Function

The CLHLS participants’ cognitive function was measured using the Chinese version of the MMSE, which tests four aspects of cognitive function: orientation, calculation, recall, and language [51]. Adapted from the international MMSE questionnaire and validated in previous studies [52,53], the Chinese version of the MMSE takes into consideration cultural and socioeconomic conditions of Chinese elderly so that all question items in the test could be easily comprehended and answered. A total of 13 question items compose the test, with total scores ranging from 0 to 30. Lower scores indicate poorer cognitive function. More details about the Chinese version of the MMSE are shown in Table 1.

#### 2.2.2. Cognitive Impairment

Following previous studies’ definition of cognitive impairment [51,52,53], a CLHLS participant who scored less than 24 in the Chinese version of the MMSE was classified as having cognitive impairment, whereas one scoring 24 or higher was classified as having no cognitive impairment.

#### 2.2.3. Socio-Demographic and Health Related Characteristics

According to the review by the international and Chinese research on cognitive impairment of the elderly, the risk factors for cognitive impairment of the elderly are divided into the following six categories in this study (see Table 2): (1) demographics, including gender, age, education, marriage, income, place of residence and coresidence; (2) psychological factors, represented by psychological well-being (PWB), which is the total score of seven questions ranging from 7 to 35, including looking on the bright side of things, keeping my belongings neat and clean, feeling fearful or anxious, feeling lonely and isolated, making my own decisions, feeling useless with age, and being happier when younger, with a higher score indicating better well-being. The specific calculation method for PWB was performed according to previous studies [54,55]; (3) lifestyle, including fresh fruit intake, fresh vegetables intake, smoking, drinking and exercising; (4) social engagement, including taking part in outdoor activities, playing cards/mahjong, and taking part in social activities; (5) ADL, which was the total score (ranging from 6 to 18) of six aspects in daily living ability, including bathing, dressing, bathroom use, indoor transferring, continence and feeding. Depending on the independence of the elderly individual in completing the above actions, they were given 1 score (complete dependence on others), 2 scores (partial independence) or 3 scores (complete independence), with a higher score indicating better daily living ability; and (6) cardiovascular risk factors, including hypertension, diabetes, heart disease, stroke or CVD, and orthopedic disease.

### 2.3. Statistical Analysis

The data were analyzed by using Stata (Stata version 14.0 for Windows, StataCorp LLP, College Station, TX, USA). Descriptive statistics were used to characterize study participants, including demographic characteristics, changes in cognitive scores from 2005 to 2014 and risk factors for cognitive impairment. One-way ANOVA was used to analyze the cognitive function of the elderly under different socio-demographic conditions in 2005, 2008, 2011 and 2014 respectively. Based on the balance panel data from 2005 to 2014, binary logistic regression analysis was used to investigate the association between cognitive impairment and six categories of risk factors. The dependent variable was cognitive impairment (with impairment = 1, without impairment = 0). The results of the binary logistic regression analysis were expressed in OR with a confidence interval of 95%, and the test level was 0.05 on both sides.

## 3. Results

### 3.1. Changes and Demographic Differences in Cognitive Function of Chinese Elderly

#### 3.1.1. Changes in the Cognitive Function of the Chinese Elderly: 2005–2014

Based on the four waves of survey data of CLHLS in 2005, 2008, 2011 and 2014, this study analyzed changes in cognitive function of the elderly in China from 2005 to 2014. To analyze changes in cognitive function of the elderly in China in more detail, this decade was divided into three phases: 2005 to 2008, 2008 to 2011 and 2011 to 2014. Changes in cognitive function of the elderly in China during these three phases were observed. At the same time, the general changes in the cognitive function of the elderly in China from 2005 to 2014 were also reported, as shown in Table 3. On one hand, the cognitive function of the elderly in China does not show a linear deterioration trend with increasing age but shows a diversified change in deterioration due to unchanged or improved cognitive function. On the other hand, the crucial detail among these data is whether in 10 years or at a certain phase in this decade (2005 to 2014), the elderly with deteriorating cognitive function account for the highest proportion. Generally, during the 2005 to 2014 decade, greater than 55% of Chinese elderly’s cognitive function deteriorated, highlighting the trend that the Chinese elderly’s cognitive function is not optimistic. Cognitive function was “unchanged” and “improved” in some of the elderly during this decade, which suggests that appropriate interventions can be utilized to improve or maintain the cognitive function of the elderly. 

#### 3.1.2. Demographic Differences of the Cognitive Function of Chinese Elderly: 2005–2014

To analyze demographic differences with respect to changes in the cognitive function of the Chinese elderly, we calculated the mean cognitive scores in different groups from 2005 to 2014, and based on the mean cognitive scores in 2005, we calculated the decrease in cognitive scores in 2008, 2011 and 2014 (results are shown in Table 4). Overall, whether the mean cognitive scores of the Chinese elderly in each wave survey or the decline in mean cognitive scores is used, there are significant differences in terms of gender, age, education, marital status and place of residence.

In terms of gender differences with respect to the cognitive function of the elderly, the mean cognitive scores of the male elderly (27.87, 27.07, 26.68 and 25.52) were always higher than those of the female elderly (26.32, 25.33, 24.47 and 22.69) from 2005 to 2014; however, the decrease (2.35) in male cognitive ability was lower than in females (3.63), demonstrating that cognitive function of the female elderly was declining faster than in the male elderly. There were also significant age differences in cognitive function of the elderly from 2005 to 2014, and the mean cognitive scores in elderly people aged 86 and over (24.25, 22.30, 22.40 and 20.29) were always lower than those aged 76-85 (26.40, 26.28, 25.91 and 25.73) and those aged 64 to 75 (27.91, 27.59, 27.49 and 27.32). Moreover, the decline (9.71) in cognitive scores was greater than that in individuals aged 76 to 85 (5.94) and aged 64 to 75 (4.09). There were also obvious differences in marital status, showing that older adults with spouses exhibit much higher mean cognitive function (27.91, 27.27, 26.88 and 26.10) than adults without spouses (25.97, 25.01, 24.38 and 22.60), and the decrease level was much lower (3.37 vs. 1.81).

Elderly individuals with no formal education exhibited the lowest mean cognitive scores (25.99, 24.97, 23.93 and 22.20), followed by those with 0-6 of years of education (27.85, 27.00, 26.76 and 25.61) and those with 7 or more years of education (28.90, 28.32, 28.12 and 26.68). Moreover, the higher the education level was, the slower the decline in cognitive scores. The decreased level (2.22) of the elderly with 7 or more years of education was lower than that of those with 0-6 years of education (2.24) and those with no formal education (3.79). We observed that mean cognitive scores among urban elderly dwellers (28.05, 27.11, 26.02 and 24.15) were the highest, followed by town elderly dwellers (27.32, 25.85, 25.73 and 24.14), with the lowest being observed in rural elderly dwellers (26.70, 25.99, 25.10 and 23.85). However, the decreased level of cognitive function among urban elderly dwellers (3.90) was higher than in town (3.18) and rural elderly dwellers (2.85). On the whole, from 2005 to 2014, mean cognitive scores of the Chinese elderly decreased from 27.05 to 24.03, and the decreased level of mean cognitive function of the whole elderly population was 3.02.

### 3.2. Risk Factors for Cognitive Impairment of the Chinese Elderly

We used binary logistic regression analysis to investigate the association between cognitive impairment and the six categories of risk factors. The association between the six categories of risk factors and cognitive impairment are shown in Table 5. 

Among demographic characteristics, being female (OR = 1.41, 95%CI = 1.16–1.72, *p* < 0.001) and increasing age (OR = 1.07, 95%CI = 1.06–1.08, *p* < 0.001) were associated with higher odds for cognitive impairment. In contrast, higher education level (OR = 0.91, 95%CI = 0.88–0.94, *p* < 0.001), having a spouse (OR = 0.72, 95%CI = 0.61–0.87, *p* < 0.001) and higher income (OR = 0.93, 95%CI = 0.88–0.99, *p* = 0.016) were associated with lower odds for cognitive impairment. However, place of residence and co-residence were not associated with cognitive impairment. In addition, better psychological well-being (OR = 0.93, 95%CI = 0.91–0.95, *p* < 0.001) was associated with lower odds for cognitive impairment, and in lifestyle, we found that fresh fruit (OR = 0.81, 95%CI = 0.75–0.88, *p* < 0.001) and vegetable (OR = 0.84, 95%CI = 0.76–0.92, *p* < 0.001) intake were associated with lower odds for cognitive impairment. Surprisingly, smoking, drinking and exercising were not associated with cognitive impairment. Furthermore, social engagement, including taking part in outdoor activities (OR = 0.89, 95%CI = 0.85–0.93, *p* < 0.001), playing cards/mahjong (OR = 0.84, 95%CI = 0.79–0.91, *p* < 0.001) and social activities (OR = 0.81, 95%CI = 0.73–0.91, *p* < 0.001) were associated with lower odds for cognitive impairment. Better ADL (OR = 0.77, 95%CI = 0.72–0.81, *p* < 0.001) were also associated with lower odds for cognitive impairment. Finally, in cardiovascular risk factors, hypertension (OR = 1.22, 95%CI = 1.03–1.44, *p* = 0.018), heart disease (OR = 0.77, 95%CI = 0.61–0.98, *p* = 0.036), stroke or CVD (OR = 1.35, 95%CI = 1.03–1.77, *p* = 0.029) were associated with cognitive impairment, while diabetes and orthopedic disease were not associated with cognitive impairment.

## 4. Discussion

With the rapid increase in China’s aging population, the cognitive function of the elderly has become an important public health issue. To the best of our knowledge, the present study is the first to explore changes in cognitive function and risk factors for cognitive impairment among the Chinese elderly from a dynamic perspective. In addition, our study also explored differences in demographic characteristics of Chinese elderly in terms of cognitive function. We found that cognitive function of the elderly in China exhibits three states, namely, “deterioration”, “unchanged” and “improvement”, during the decade from 2005 to 2014, similar to previous studies [56,57]. However, the proportion of the elderly with deteriorated cognitive function was the highest, and mean cognitive score of the elderly in China decreased from 27.05 to 24.03 during this decade. This highlights the fact that the cognitive function of the elderly in China is not optimistic.

This study uses one-way ANOVA to analyze the demographic characteristics of Chinese elderly from the perspective of cognitive function. Previous studies in other countries or regions found that the cognitive function score of the male elderly was higher than that of the female elderly, and the female elderly tended to experience severer cognitive impairment [12,26], which our study supports. Furthermore, our study reaffirms that the cognitive function of the oldest elderly (aged 86 and over) decreased more quickly than that of the younger age groups (aged 64 to 75 and aged76 to 85) from 2005 to 2014, and the cognitive function aged 64 to 75, 76 to 85 and 86 and over in China was worse than that in other developed countries or regions [57,58]. Our study found that the elderly with higher education levels experienced lower decreases in cognitive function, which is consistent with previous studies in other countries [27,29]. Our study is the first to identify that the cognitive function among the city, town and rural elderly declined at different rates, among which the city elderly declined the fastest. These findings provide important insights into the implementation of cognitive function interventions for different older people.

In China, previous literature has extensively documented risk factors for cognitive impairment, but they are scattered. Our study is the first to analyze these risk factors in a framework from a comprehensive perspective. We summarize these risk factors from six aspects, including demographic characteristics, psychological factors (represented by PWB), lifestyle, social engagement, ADL, and cardiovascular risk factors. In terms of demographic characteristics, some of our findings support previous studies, such as gender, age, marriage and income being significantly associated with cognitive impairment. Place of residence and coresidence had no relationship with cognitive impairment. A previous study found that elderly living with family tended to have better cognitive function than those living in nursing homes [18], in contrast to our study. One explanation for this discrepancy is that most Chinese elderly lived with family members, which may lead to imbalances in study samples. We observed that PWB, increased social engagement and better ADL were significantly associated with cognitive impairment, highly coincident with previous studies.

Compared to studies in other developed countries, the most controversial results we observed fell into the categories of lifestyle and cardiovascular risk factor. We observed a significant correlation between the intake of fresh fruit and vegetable and the reduced risk of cognitive impairment, which is supported by previous studies. However, our findings concerning smoking, drinking and exercising were not consistent with previous studies. Previous studies have also found no significant correlation between smoking and cognitive impairment. Some previous studies observed that drinking is associated with cognitive impairment, while others have denied this relationship [59]. Some previous literature also found no benefit of exercise on cognitive function, while others found the evidence [60,61]. One possible explanation for this discrepancy is that lifestyle factors, including smoking, drinking and exercising, are easily changed and are not consistent over long periods of time. Another possibility is that individuals have different levels of exposure to cigarette and alcohol. Further studies should explore the mechanism of smoking on cognitive impairment by investigating the number of years of smoking and drinking to control these factors. In terms of cardiovascular risk factors, previous studies indicated that midlife cardiovascular risk factors increase the risk for cognitive impairment in later years [32]; however, studies assessing the relationship between cardiovascular risk factors and cognitive impairment in later years are inconsistent. We observed no significant correlation between cognitive impairment and hypertension, heart disease, stroke or CVD, diabetes and orthopedic disease. Future study should examine the precise link between cardiovascular risk factors and cognitive impairment.

There were some limitations in this study. First, the Chinese version of MMSE has certain defects, as it does not reflect ecological value when measuring cognitive function. Also, there is no reliable change index based on MMSE due to the limitations of data. Of course, these two points are of great significance for the accuracy in cognitive function of the elderly. Second, due to limited data, we cannot analyze the impact of environmental pollution, genetic factors on cognitive impairment in Chinese elderly. In fact, previous studies have indicated that these are key risk factors for cognitive impairment in the elderly. Third, since CLHLS does not distinguish cognitive impairment, dementia and AD, we have failed to provide more information about the relationship between cognitive impairment, dementia and AD of Chinese elderly.

## 5. Conclusions

With the rapidly increasing aging population, the cognitive function of the elderly in China is not optimistic, which poses a huge challenge for the public health system and the care system in China. Our study provides a basis for understanding the changes in cognitive function and the risk factors of cognitive impairment in the elderly in China. Future research on cognitive function of the elderly in China should consider the use of measuring tools covering ecological values, fully considering the impact of social and ecological environment, genetic and other factors on cognitive function of the elderly. In addition, the relationship between cognitive function, dementia, AD should also be studied.

The results of this study have important policy implications for the intervention of cognitive function in Chinese elderly. First, the monitoring system of cognitive function changes and risk factors in the elderly should be established. In clinical practice, prevention, intervention, treatment and care of cardiovascular and cerebrovascular diseases in the elderly should be strengthened. In medical research, special medical research project on cerebrovascular disease should be set up to promote precise medical research on cerebrovascular diseases, focusing on precise medical cohort research on cerebrovascular disease, pharmacogenomics research on cerebrovascular disease, and big data research on cerebrovascular disease. Second, health education should be carried out to improve health awareness and self-health management ability of the elderly, in view of their unhealth behaviors such as smoking, drinking and lack of exercise. At the same time, the role of primary health care service institutions in health promotion and health management of the elderly should be strengthened. Third, nonpharmacological treatments such as psychotherapy and cognitive behavioral therapy, which can help delay the occurrence of cognitive impairment in the elderly, should be actively promoted. Finally, China should establish a long-term care system as soon as possible to provide high-quality and efficient care services for those with cognitive impairment. Also, support services (respite care, emotional support, stress relief, etc.) should be provided to family members caring for the elderly with cognitive impairment.

## Figures and Tables

**Table 1:** The Chinese version of the mini-mental state examination adopted in the CLHLS.

Domain	Question	Score (Total = 30)
Orientation	What time of day is it right now (morning, afternoon, evening)?	1
	What is the animal year of this year?	1
	What is the date (day and month) of the mid-autumn festival?	1
	What is the season right now?	1
	What is the name of this county or district?	1
Naming foods	Please name as many kinds of food as possible in 1 minute (1 point for each food and 7 points for those who name 7 or more foods).	7
Registration	Table, apple, cloth. Please repeat these 3 objects.	3
Attention and calculation	I will ask you to spend $3 from $20, then you must spend $3 from the number you arrived at and continue to spend $3 until you are asked to stop.	5
Copy a figure	The individual is asked to draw a figure of overlapping pentagons.	1
Recall	Name the 3 objects learned earlier (table, apple, and cloth).	3
Language	Naming pen and watch.	2
	Repeating the following sentence: “What you plant is what you will get.”	1
	The individual is asked to follow the interviewer’s instruction: “Take the paper using your right hand, fold it in the middle using both hands, and place the paper on the floor.”	3

**Table 2:** Descriptive statistics for study sample

Variables	Measurement	2005 (%)	2008 (%)	2011 (%)	2014 (%)	*p*-Value
Gender	Male = 0	47.06%	47.06%	47.06%	47.06%	<0.001
	female = 1	52.94%	52.94%	52.94%	52.94%	
Age	64 to 75 = 1	60.85%	46.75%	30.46%	13.17%	<0.001
	76 to 85 = 2	26.77%	34.69%	44.41%	51.71%	
	86 and over = 3	12.37%	18.55%	25.12%	35.11%	
Education (years)	No formal education = 1	50.71%	50.71%	50.71%	50.71%	<0.001
	1 to 6 = 2	35.69%	35.69%	35.69%	35.69%	
	7 or more = 3	13.60%	13.60%	13.60%	13.60%	
Marriage status	Without spouse = 0	44.30%	49.56%	54.43%	59.26%	<0.001
	Have spouse = 1	55.70%	50.44%	45.57%	40.74%	
Income (yuan)	Continuous measurements	2374 (1.03)	2412 (1.15)	2253 (1.30)	2347 (1.31)	<0.001
Place of residence	City =1	17.13%	16.98%	20.28%	21.21%	<0.001
	Town = 2	18.86%	23.01%	35.42%	38.80%	
	Rural = 3	64.00%	60.01%	44.30%	39.99%	
Coresidence	Living with family = 1	86.05%	83.17%	82.20%	81.26%	<0.001
	Living alone = 2	12.87%	15.44%	16.07%	16.55%	
	Living in nursing home = 3	1.08%	1.38%	1.73%	2.19%	
PWB	Continuous measurements	26.38(4.04)	25.97(3.79)	27.02(4.06)	26.50(4.15)	<0.001
Fresh fruit intake	Rarely or never = 1	23.67%	20.28%	28.04%	29.55%	<0.001
	Sometimes = 2	40.57%	39.99%	34.96%	34.05%	
	Often = 3	24.74%	26.93%	21.69%	21.88%	
	Almost every day = 4	11.03%	12.79%	15.31%	15.52%	
Fresh vegetable intake	Rarely or never = 1	1.58%	1.38%	2.50%	3.97%	<0.001
	Sometimes = 2	8.91%	7.07%	6.11%	7.12%	
	Often = 3	33.38%	23.74%	25.74%	26.53%	
	Almost every day = 4	56.13%	67.81%	65.66%	62.38%	
Smoking	Past and present = 1	22.65%	18.42%	18.05%	15.12%	<0.001
	Past or present = 2	16.49%	20.76%	20.28%	23.11%	
	Rarely or never = 3	60.86%	60.82%	61.66%	61.77%	
rinking	Past and present = 1	32.65%	25.38%	26.30%	22.27%	<0.001
	Past or present = 2	13.35%	18.54%	17.87%	18.94%	
	Rarely or never = 3	54.00%	56.08%	55.84%	58.79%	
Exercising	Past and present = 1	22.69%	18.92%	19.45%	16.03%	<0.001
	Past or present = 2	24.62%	36.19%	40.49%	36.90%	
	Rarely or never = 3	52.69%	44.88%	40.06%	47.07%	
Outdoor activity	Rarely or never = 1	19.98%	23.43%	26.05%	35.08%	<0.001
	Sometimes = 2	6.65%	5.03%	5.57%	4.28%	
	Monthly = 3	4.88%	4.30%	2.23%	2.58%	
	Weekly = 4	12.45%	12.29%	11.37%	9.98%	
	Daily = 5	56.05%	54.94%	54.78%	48.07%	
Playing Cards or Mahjong	Rarely or never = 1	73.88%	76.87%	79.42%	81.07%	<0.001
	Sometimes = 2	5.53%	4.61%	3.92%	3.43%	
	Monthly = 3	3.76%	2.96%	2.00%	2.35%	
	Weekly = 4	8.28%	5.65%	5.62%	5.36%	
	Daily = 5	8.57%	9.91%	9.04%	7.79%	
Social activities	Rarely or never = 1	79.63%	82.64%	79.18%	81.18%	<0.001
	Sometimes = 2	10.26%	6.99%	10.62%	9.53%	
	Monthly = 3	3.11%	4.23%	2.89%	2.82%	
	Weekly = 4	2.81%	2.38%	2.96%	2.35%	
	Daily = 5	4.19%	3.76%	4.35%	4.13%	
ADL	Continuous measurements	17.91 (0.63)	17.88 (0.80)	17.58 (1.42)	17.03 (2.43)	<0.001
Hypertension	No = 0	78.67%	76.26%	66.52%	62.18%	0.703
	Yes = 1	21.33%	23.74%	33.48%	37.82%	
Diabetes	No = 0	97.33%	96.66%	94.82%	92.77%	0.741
	Yes = 1	2.67%	3.34%	5.18%	7.23%	
Heart disease	No = 0	90.59%	89.73%	85.46%	83.45%	0.020
	Yes = 1	9.41%	10.27%	14.54%	16.55%	
Stroke or CVD	No = 0	95.32%	93.92%	91.42%	88.66%	<0.001
	Yes = 1	4.68%	6.08%	8.58%	11.34%	
Orthopedic disease	No = 0	91.87%	98.12%	95.29%	95.68%	<0.001
	Yes = 1	8.13%	1.88%	4.71%	4.32%	

Note: Results are expressed as a percentage of categorical data and as the mean (standard deviation) for continuous data; the *p*-value was calculated by the chi-square test.

**Table 3:** Changes in cognitive function of the Chinese elderly: 2005–2014.

Phase/Year		Deterioration	Unchanged	Improvement
Phase 1	2005–2008	1185 (45.52%)	534 (20.51%)	884 (33.96%)
Phase 2	2008–2011	1154 (44.33%)	475 (18.25%)	974 (37.42%)
Phase 3	2011–2014	1225 (47.06%)	512 (19.67%)	866 (33.27%)
Overall	2005–2014	1434 (55.09%)	448 (17.21%)	721 (27.70%)

**Table 4:** Cognitive function of different groups from 2005–2014.

Characteristic Variables	2005			2008			2011			2014		
		F	*p*		F	*p*		F	*p*		F	*p*
Male	27.87 ^a^	92.19	<0.001	27.07	64.23	<0.001	26.68	87.14	<0.001	25.52	87.57	<0.001
	3.50 ^b^			4.91			5.22			6.87		
				(−0.8) ^c^			(−1.19)			(−2.35)		
Female	26.32			25.33			24.47			22.69		
	4.58			6.00			6.68			8.38		
				(−0.99)			(−1.85)			(−3.63)		
64 to 75	27.91	124.95	<0.001	27.59	176.25	<0.001	27.49	141.76	<0.001	27.32	190.42	<0.001
	3.13			4.01			3.54			4.09		
				(−0.32)			(−0.42)			(−0.59)		
76 to 85	26.40			26.28			25.91			25.73		
	4.42			4.72			5.51			5.94		
				(−0.12)			(−0.49)			(−0.67)		
86 and over	24.25			22.30			22.40			20.29		
	6.19			8.12			8.15			9.71		
				(−1.95)			(−1.85)			(−3.96)		
Have spouse	27.91	147.59	<0.001	27.27	111.74	<0.001	26.88	112.79	<0.001	26.10	130.92	<0.001
	3.18			4.33			4.66			6.04		
				(−0.64)			(−1.03)			(−1.81)		
Without spouse	25.97			25.01			24.38			22.60		
	4.96			6.43			6.89			8.57		
				(−0.96)			(−1.59)			(−3.37)		
No formal education	25.99	101.15	<0.001	24.97	72.27	<0.001	23.93	104.94	<0.001	22.20	83.24	<0.001
	4.60			6.15			6.86			8.50		
				(−1.02)			(−2.06)			(−3.79)		
1 to 6	27.85			27.00			26.76			25.61		
	3.61			4.92			5.18			6.61		
				(−0.85)			(−1.09)			(−2.24)		
7 or more	28.90			28.32			28.12			26.68		
	2.42			3.60			3.28			6.43		
				(−0.58)			(−0.77)			(−2.22)		
Urban	28.05	19.97	<0.001	27.12	8.15	<0.001	26.02	5.01	0.006	24.15	0.45	0.636
	3.01			4.94			6.37			8.20		
				(−0.93)			(−2.03)			(−3.9)		
Town	27.32			25.85			25.73			24.14		
	24.14			5.45			5.87			7.55		
				(−1.47)			(−1.59)			(−3.18)		
Rural	26.70			25.99			25.10			23.85		
	4.39			5.78			6.21			7.90		
				(−0.7)			(−1.6)			(−2.8)		
Whole	27.05			26.15			25.51			24.03		
	4.18			5.58			6.13			7.83		
				(−0.9)			(−1.5)			(−3.0)		

Note: The *p* value was calculated by one-way ANOVA analysis; ^a^ mean of the cognitive scores; ^b^ standard error of the cognitive scores; ^c^ decreased level based on the level of 2005.

**Table 5:** Binary logistic regression analysis of risk factors for cognitive impairment in the elderly.

Variables	Odds Ratio	SE	z-Values	*p*-Values	95%CI
Gender	1.41	0.14	3.50	<0.001	1.16–1.72
Age	1.07	0.01	15.52	<0.001	1.06–1.08
Education	0.91	0.01	−6.35	<0.001	0.88–0.94
Marriage	0.72	0.06	−3.58	<0.001	0.61–0.87
Income	0.93	0.03	−2.40	0.016	0.88–0.99
Place of residence	0.91	0.05	−1.74	0.081	0.82–1.01
Coresidence	0.94	0.09	−0.68	0.495	0.78–1.13
PWB	0.93	0.01	−7.51	<0.001	0.91–0.95
Fresh fruit intake	0.81	0.03	−5.03	<0.001	0.75–0.88
Fresh vegetable intake	0.84	0.04	−3.76	<0.001	0.76–0.92
Smoking	1.11	0.08	1.39	0.166	0.96–1.28
Drinking	0.92	0.06	−1.21	0.228	0.81–1.05
Exercising	0.98	0.06	−0.29	0.770	0.88–1.10
Outdoor activities	0.89	0.02	−5.44	<0.001	0.85–0.93
Play cards/mahjong	0.84	0.03	−4.50	<0.001	0.79–0.91
Social activities	0.81	0.04	−3.68	<0.001	0.73–0.91
ADL	0.77	0.02	−9.05	<0.001	0.72–0.81
Hypertension	1.22	0.10	2.36	0.018	1.03–1.44
Diabetes	1.07	0.20	0.35	0.730	0.74–1.54
Heart disease	0.77	0.09	−2.10	0.036	0.61–0.98
Stroke or CVD	1.35	0.19	2.18	0.029	1.03–1.77
Orthopedic disease	0.81	0.16	−1.07	0.284	0.55–1.19

## Abbreviations

The following abbreviations are used in this manuscript:

AD	Alzheimer’s disease
ADL	Activities of daily living
CLHLS	Chinese Longitudinal Healthy Longevity Survey
CVD	Cerebrovascular diseases
MMSE	Mini-mental state examination
PWB	Psychological well-being
